# Food-Based Dietary Guidelines around the World: A Comparative Analysis to Update AESAN Scientific Committee Dietary Recommendations

**DOI:** 10.3390/nu13093131

**Published:** 2021-09-08

**Authors:** Montaña Cámara, Rosa María Giner, Elena González-Fandos, Esther López-García, Jordi Mañes, María P. Portillo, Magdalena Rafecas, Laura Domínguez, José Alfredo Martínez

**Affiliations:** 1 Nutrition and Food Science Department, Pharmacy Faculty, Complutense University of Madrid (UCM), Plaza Ramón y Cajal, s/n, 28040 Madrid, Spain; ladoming@ucm.es; 2 Department of Pharmacology, Faculty of Pharmacy, University of Valencia, 46100 Burjassot, Spain; Rosa.M.Giner@uv.es; 3 Department of Food Technology, CIVA Research Center, University of La Rioja, Madre de Dios, 26006 Logroño, Spain; elena.gonzalez@unirioja.es; 4 Department of Preventive Medicine and Public Health, School of Medicine, Universidad Autónoma de Madrid, Instituto de Investigación Sanitaria Hospital Universitario La Paz (IdiPAZ), CIBER of Epidemiology and Public Health (CIBERESP), 28029 Madrid, Spain; esther.lopez@uam.es; 5 Department of Preventive Medicine and Public Health, Food Sciences, Toxicology and Forensic Medicine, Faculty of Pharmacy, University of Valencia, 46100 Burjassot, Spain; jordi.manes@uv.es; 6 Nutrition and Obesity Group, Department of Pharmacy and Food Sciences, University of the Basque Country (UPV/EHU) and CIBERobn Physiopathology of Obesity and Nutrition, Institute of Health Carlos III, and BIOARABA Health Research Institute, 01006 Vitoria-Gasteiz, Spain; mariapuy.portillo@ehu.eus; 7 Departament de Nutrition, Food Science and Gastonomy, Faculty of Farmacy and Food Science, University of Barcelona, 08028 Barcelona, Spain; magdarafecas@ub.edu; 8 IMDEA + CSIC/UAM y CIBEROBN, 28049 Madrid, Spain; jalfredo.martinez@imdea.org

**Keywords:** food-based dietary guidelines, national dietary recommendations, healthy eating, health promotion, public health

## Abstract

Food-Based Dietary Guidelines (FBDG) include dietary recommendations based on food groups according to the general and accepted nutrition principles and current scientific evidence. Adoption of FBDG contributes to the prevention of malnutrition in all its forms, promotes human health, and reduces environmental impact. The present review aims to perform an international comparative analysis of the FBDG adopted in different countries from three different continents (America, Asia, and Europe), with particular reference to the Spanish Food Safety and Nutrition Agency (AESAN, Agencia Española de Seguridad Alimentaria y Nutrición) Scientific Committee dietary recommendations. A total of twelve countries with the most updated FBDG and/or closest to the traditional and cultural preferences of Spain were finally selected. All the reviewed FBDG provided recommendations for fruits, vegetables, cereals, legumes, nuts, milk and dairy products, meat and derivatives, fish, eggs, water, and oil; however, remarkable differences regarding recommended amounts were found among countries.

## 1. Introduction

Foods are sources of macronutrients (carbohydrates, proteins, fats) and micronutrients (vitamins and minerals) necessary to cover human metabolism requirements for life and survival, concerning energy demands for sustainability, growth, development, and reproduction [1]. In addition, foods contain biologically active components called “bioactive compounds” or “phytochemicals”, which are able to contribute to achieving an adequate health status [2].

The knowledge of food composition (nutrients and bioactive compounds) as well as defining the different food groups, where they are categorized, is fundamental for food intake decision-making and promoting human health [1,3]. The consumption of foodstuffs from different food groups in balanced proportions ensures the adequate intake of nutrients and bioactive compounds with potential benefits for human health [4]. Scientific evidence has demonstrated that a varied and balanced diet with a predominant consumption of plant-based food groups (fruits, vegetables, legumes, cereals, nuts, etc.) over food groups of animal origin promotes health and reduces environmental impact [5]. According to EAT-Lancet Commission, the adoption of healthy and sustainable diets could reduce 19.0–23.6% of global deaths (10.8–11.6 million) every year [6]. Thus, focusing exclusively on human health is not sufficient as inadequate eating patterns adversely affect the environment and jeopardize well-being, quality of life, and survival of present and future generations [7,8].

In general, dietary recommendations are based on Dietary Reference Values (DRV) (also named Recommended Nutrient Intake, RNI; or Recommended Dietary Allowances, RDAs), which refer to specific nutrients and their recommended intake for each particular group of the population in order to avoid nutritional deficiencies that can compromise health. One example is Spain, where the Scientific Committee of the Spanish Agency for Food Safety and Nutrition (AESAN) in 2019 approved a scientific report to update DRVs of 15 minerals and 13 vitamins for the Spanish population [9].

The European Food Safety Authority (EFSA) has encouraged Member States of the European Union (EU) to establish dietary recommendations focused on food groups based on DRVs [10]. In this context, The World Health Organization (WHO) and the Food and Agriculture Organization of the United Nations (FAO) have promoted the development of national food-based dietary recommendations in line with the Sustainable Healthy Diets; that is, considering the economic, cultural, social, and environmental conditions of each country [11].

In this context, Food-Based Dietary Guidelines (FBDG) are considered short messages expressed as dietary recommendations based on food groups, in accordance with the general and accepted nutrition principles and current scientific evidence [7]. FBDG are focused on preventing malnutrition in all its forms, promoting human health, as well as reducing environmental impact through the incorporation of the concept of sustainability within the healthy diet definition [7,12,13].

The objective of the present review is to perform an international comparative analysis of the FBDG adopted in different countries from three different continents (America, Asia, and Europe), with particular reference to the AESAN (Agencia Española de Seguridad Alimentaria y Nutrición) Scientific Committee dietary recommendations.

## 2. Materials and Methods

Different countries have established FBDG. On its website (http://www.fao.org/nutrition/education/food-dietary-guidelines/en/, accessed on 6 September 2021), FAO [14] offers a general description of food guides that include recommendations for approved food groups in diverse countries. Inclusion criteria established to select FBDG subjected to this review include the most updated dietary guidelines as well as those that are closest to the traditional and cultural preferences of Spain.

Twelve countries were selected considering the inclusion criteria. As shown in Table 1, the present work critically reviews the international food guides of the United States, China, the Nordic countries (Finland, Norway, and Sweden), the United Kingdom, Germany, the Netherlands, France, Portugal, Italy, and Spain.

## 3. Results and Discussion

In general, all the national and international dietary guidelines selected and reviewed in the present work were in accordance with the Sustainable Healthy Diets (healthy eating models with a low environmental impact) [29], as they provided the following recommendations:

- A varied, balanced diet: eat a wide range of food products from different food groups and maintain an adequate balance between the energy ingested and the expenditure of the individuals.
- A diet based mainly on plant-based food; that is, fruits, vegetables, minimally processed tubers, cereals (preferably whole grains), unsalted seeds and nuts, as well as oils and fats with a beneficial omega 3:6 ratio.
- Moderate consumption of meat and its derivatives and dairy products. The diet should incorporate small amounts of fish and aquatic products from certified fisheries.
- Very limited consumption of food rich in fats, simple sugars, or salt, as well as low in micronutrients (e.g., fried potatoes, confectionery products, sugary drinks, etc.).
- Intake of water as the main drink, limiting the consumption of other drinks, particularly sugary soft drinks.

### 3.1. Illustrations Included in the Selected Food Guides

The most commonly used graphic representation in the reviewed dietary guidelines was an illustration with comprehensible and clear key messages and traffic light colors: green, amber/yellow, and red. The first one (green color) recommends an increase of the consumption of certain food groups, above all plant-based food (fruits, vegetables, legumes, etc.). The amber color promotes a replacement of one specific food group by a better option (e.g., refined cereals by whole grains). Finally, the red color recommends a reduction in the consumption of particular food groups, generally red and processed meat, salt, sugar, and alcohol. The dietary guidelines of Sweden, The Netherlands, France, and Spain (GENCAT, Generalitat de Catalunya) used this graphic representation [19,22,23,28].

The nutritional circle, oval, or wheel as well as the plate were the next illustrations most often used in the selected dietary guidelines. Germany, the United Kingdom, and Portugal used the nutritional circle, oval, and wheel, respectively [20,21,24], whereas the United States, China, and Finland included the plate model in their food guides [15,16,17]. Except for the Finnish model, both types of graphic representations are divided into segments with a different size, which reflects how different food groups should contribute towards the total diet. Although both illustrations depict the relative weight of each food group, the nutritional circle, oval, and wheel refer to the whole diet, while the plate represents a single meal.

The pyramid model was used in the dietary guidelines of Finland and Spain (AESAN) and it is divided in segments or steps according to the contribution of each food group to the whole diet, so that the lower the level (close to the bottom level), the higher the importance in the diet [17,26,27].

The Chinese and Swedish food guides included other illustrations not used in other countries, such as the pagoda and the abacus as well as the keyhole symbol, respectively [16,19]. The pagoda model is similar to the pyramid as the most important food groups are located in the lower levels. The abacus is directed to children (8–11 years old) and it contains 6 rows with colored beads. Each food group is represented by a specific color, and the number of beads in each row indicates the portions recommended [16]. The Italian food guides do not include graphic representations [25].

Table 2 provides a summary of the illustrations used in the selected food guides.

Herforth et al. carried out an interesting review of the current FBDG whose information was available in the repository of the FAO in order to evaluate the similarities and differences in the key nutrition messages among countries, although they do not include information about FBDG that use key messages as graphic representations of the food groups [30]. In the present manuscript, a total number of 90 FBDG from different regions of the world were finally selected and reviewed: Europe (33 FBDG), Latin America and the Caribbean (27), Asia and the Pacific (17), Africa (7), Near East (4), and North America (2). The results revealed that the illustrations most commonly used in the above-mentioned FBDG were the pyramid and the circle or plate (39.7% and 26.9%, respectively). In addition, this review was mainly focused on the Spanish dietary recommendations.

Soy milk in the food group “dairy products” (milk, yogurt, cheese, etc.) as an alternative of the above-mentioned products was only considered in the United States and the United Kingdom. In addition, Norway was the only country that specifically recommended the consumption of lean dairy products.

### 3.2. Comparative Analysis of the Food Groups

The results from the comparative analysis of the selected national and international food guides carried out by the authors of the present work are summarized in Table 3.

Focusing on plant-based food groups, it is important to highlight that the dietary guidelines from Finland, Norway, Italy, and Spain (GENCAT) specifically recommended the consumption of whole grains. In the rest of the countries (United States, China, Sweden, United Kingdom, Germany, The Netherlands, France, Portugal, and Spain (AESAN)), it was indicated that the consumption of whole products should be promoted versus the consumption of refined ones. With respect to the soy products, advised consumption was only recommended in the food guides of the United States and China. 

Regarding the food groups of animal origin, all the selected dietary guidelines generally recommended reducing the consumption of meat, mainly red and/or processed meat. The inclusion of seafood within the food group “fish” was only considered in the food guides of the United States, Sweden, Italy, and Spain (GENCAT).

The consumption of virgin olive oil was particularly promoted in some of the countries, except for the United States, China, The Netherlands, France, and Portugal. All the reviewed dietary guidelines generally recommended reducing the intake of salt, simple sugars, and alcohol, but only few countries suggested a maximum daily intake. Finally, the performance of daily physical activity was promoted in all countries.

These results are in accordance with the studies by Montagnese et al. and Herforth et al. [30,31]. Montagnese et al. performed a comprehensive comparative analysis of the FBDG of 34 European countries. Although important differences among these dietary guidelines were found due to geographical, social, and cultural factors, the great majority of the key nutritional points were similar among countries (e.g., promotion of the consumption of plant-based food such as fruits, vegetables, and cereals, and reduction of the consumption of animal products, fats, sugars, and salt). Likewise, Herforth et al. reported that most of the reviewed food guides recommended following a varied diet, with a clear predominance of plant-based food groups (fruits, vegetables, legumes) and a limited consumption of fat, salt, and sugar. The recommendations related to nuts, dairy products, red meat, and oil were more variable among countries. In addition, the consumption of nuts, whole grains, and healthy fats was not promoted in all the countries despite the WHO recommendations.

### 3.3. Recommendations for Specific Food Groups: Established Daily Intakes

A more comprehensive comparative analysis of the selected food guides regarding the specific recommendations established for each food group was carried out. Table 4, Table 5 and Table 6 summarize the most important findings of all the reviewed dietary guidelines (see Table 1 for references). It is important to highlight that the Italian dietary guidelines establish various daily and weekly intakes for each food group according to three different caloric intakes (1500, 2000, and 2500 kcal/day). In the present work, Italian recommendations will be provided as a range of the three caloric intakes.

Fruits and vegetables are low-energy density foods whose regular consumption contributes to a diversified and nutritious diet by providing a wide range of micronutrients, such as minerals (mainly magnesium and potassium) and water-soluble vitamins (C and B_9_ or folic acid), and bioactive compounds, when consumed fresh and uncooked. Dietary fiber is another important bioactive compound in these food groups, with a general predominance of the soluble fraction (pectins, gums, mucilages) in fruits. Carotenoids without provitamin-A activity are present in some fruits and vegetables; for instance, lycopene (above all in watermelon and tomato) and zeaxanthin (especially in corn), among others [1].

Cereals, particularly whole grains, contain high quantities of insoluble dietary fiber (cellulose, hemicellulose, lignin), whereas the soluble fraction of fiber is present in significant amounts in oat. An adequate daily intake of cereals also provides soluble and insoluble carbohydrates, B-group vitamins such as B_1_, B_3_, B_6_, and B_9_, and minerals such as magnesium, zinc, calcium, and iron (especially in whole grains) [1].

Legumes are foods high in carbohydrates, fiber, and protein, with an important nutritional value. Starch, stachyose, and raffinose represent the main carbohydrates in these plant-based foods. Except for methionine (present in grains), legumes contain all amino acids, including lysine, which is limited in cereals. For this reason, it is recommended to combine the consumption of legumes and cereals in the same meal to increase its nutritional value. Other important compounds present in legumes are vitamins (B_1_, B_2_, B_3_, B_9_, C, carotenes) and minerals (calcium, magnesium, zinc, potassium, iron). Fruits, vegetables, and cereals’ fat content is low. Legumes have a proper lipid profile as monounsaturated and polyunsaturated fatty acids are the predominant ones [1,32,33].

Nuts (walnuts, almonds, hazelnuts, peanuts) are high-energy foods due to their important quantity of fat (nearly 50%); however, the lipid profile is adequate as nuts contain mostly monounsaturated and polyunsaturated fatty acids, such as linoleic acid. The consumption of nuts also provides some vitamins (B_6_, E) and minerals (magnesium, potassium) [1,3,34].

Focusing on the plant-based food groups (Table 4), all the reviewed dietary guidelines, with the exception of the Swedish one, suggested specific recommendations for fruits and vegetables. Norway, the United Kingdom, Germany, France, Italy, and Spain (AESAN and GENCAT) recommended eating at least 5 servings/day. The Portuguese food guide proposed a higher number of servings, as it recommended 3–5 servings/day for fruits and 3–5 servings/day for vegetables; that is, a total of 6–10 servings/day counting both food groups. China, Finland, and The Netherlands directly suggested specific amounts (at least 200 g/day), instead of servings. In summary, all the selected dietary guidelines promote the consumption of a great variety of fruits and vegetables, which contribute to the maintenance of an appropriate health status. According to López-González et al., the higher the quantity and variety in fruit and vegetables’ consumption, the better nutrient adequacy and diet quality [35].

Regarding the starchy foods (cereals, preferably whole grains, bread, pasta, rice, and potatoes), Portugal proposed the highest amounts (4–11 servings/day) as well as the best-described recommendations for this group by indicating household measurement for each food product and its equivalent in grams. Italy established different servings for each food item (daily amounts for bread and pasta/rice, and weekly intakes for breakfast cereals and potatoes), France 1 portion/meal, and the Chinese, Dutch, and the Italian guidelines provided a specific daily amount for starchy foods.

The food group with the lowest number of specific recommendations was soy products (only two food guides: the United States and China), followed by nuts (five countries: the United States, The Netherlands, France, Italy, and Spain (GENCAT)) and legumes (six food guides: the United States, China, Portugal, Italy, and Spain (AESAN and GENCAT)). Spain only established the frequency for legumes’ consumption (2–3 times/week in AESAN food guide and 3–4 times/week in GENCAT food guide) but not a specific weekly amount.

Finally, it is important to underline that the only country that provided specific recommendations for all the plant-based food groups was the United States, where they are expressed as cup-equivalents (c-eq) and/or ounce-equivalents (oz-eq), where 1 ounce = 0.25 cup = 29.6 mL.

Regarding recommendations for foods of animal origin, most of the dietary guidelines include milk, dairy products, fish, eggs, meat, and derivatives.

Milk is considered the most complete food item in terms of nutritional composition as it contains all essential nutrients for humans except for dietary fiber, vitamin C, and iron. Dairy products (yogurt, cheese, etc.) are an excellent option to achieve an adequate intake of certain nutrients such as calcium, phosphorus, retinol, vitamin D, and vitamin B_12_ [1].

Fish are high-protein foods with important quantities of fat (polyunsaturated fatty acids) in oily fish such as salmon, tuna, mackerel, sardines, and herring, among others. Liver fish or blue fish are good sources of vitamin D for humans. Eicosapentaenoic and docosahexaenoic acids (EPA and DHA), other vitamins (retinol, B_12_), and minerals (calcium, potassium, zinc, iron, phosphorus, iodine, selenium) are present in fish as well [1,32,33].

Eggs contain important quantities of proteins of high biological value (albumin, ovovitellin, etc.), vitamins D, E, B_2_, and B_12_, retinol, iron, and iodine. Water and proteins are mainly contained in the egg white, whereas the yolk is high-fat (cholesterol) and has significant amounts of vitamin D [1,3,34].

Meat and its derivatives are high-protein and high-fat foods with important quantities of iron, zinc, retinol, and vitamins B_1_, B_2_, B_3_, B_6_, and B_12_; however, the lipid profile (in terms of fatty acids) is not entirely adequate and lean meat is preferred. In addition, the content of vitamins E and K is very low, and they do not contain dietary fiber, carbohydrates (except for glycogen and lactose in processed meat), or vitamin C [1].

With regard to the recommended intake of foods of animal origin (Table 5), the United States, France, and Portugal provided similar recommendations for dairy products (3 cup-equivalents/day, 2 servings/day, and 2–3 servings/day, respectively). The Chinese, Finnish, and Italian dietary guidelines recommended specific amounts for milk (300 g/day, 5–6 dL/day, and 125–375 mL/day, respectively) and for cheese, in the case of Finland (2–3 slices of low-fat cheese/day) and Italy (300 g cheese/week). Norway, Sweden, the United Kingdom, Germany, The Netherlands, and Spain have no specific recommendations about daily intakes for dairy products. Spain (GENCAT) only established the frequency for dairy products’ consumption (1–3 times/day) but not a specific daily amount. The weekly recommendations for meat and its products, as well as for fish, were quite equal in China, Finland, Sweden, and France (<500–525 g meat/week). China was the only country that directly recommended the consumption of poultry or lean meat, and Italy provided different weekly servings for lean and red meat. Norway, the United Kingdom, Germany, and The Netherlands did not include specific amounts for fish, meat, and its products in their national dietary guidelines. Food groups with the least number of recommendations were seafood and eggs. Out of the thirteen selected dietary guidelines, only one (United States food guide) included one specific recommendation for seafood (8 oz-eq/week) and four for eggs (United States, China, Portugal, and Italy). Spain (GENCAT) only established the frequency for eggs’ consumption (3–4 times/week). Last but not least, the dietary guidelines of the United States, Portugal, and Italy established one specific recommendation that covers several food groups; that is, 26 oz-eq/week for meats, poultry, and eggs (United States), 1.5–4.5 servings/day for meat and fish (raw meat/fish = 30 g; cooked meat/fish = 25 g) (Portugal), and 2–3 servings/week for fish and seafood (1 serving = 150 g) (Italy).

Water is fundamental for organism survival as it regulates temperature, protects tissues and organs, allows nutrient absorption and transportation to cells, and other vital bodily functions. For that reason, all national dietary guidelines selected in the present study established recommendations related to water consumption, at least 1.2 L/day. The recommendations of water and other liquids’ consumption were similar among countries. China, the United Kingdom, Germany, Italy, and Spain (GENCAT) suggested a daily consumption of at least 1.2 L/day; that is, between 5 and 8 cups/glasses of water. However, it is important to highlight that in The Netherlands, the recommended daily intake was established in just 3 cups of tea/day. This fact demonstrates once again the strong influence of the cultural, social, and geographical factors.

Olive oil is one of the pillars of the Mediterranean Diet. It consists primarily of oleic acid (C18:1, ω-9, cis) (80–90%), fatty acids such as linoleic acid (C18:2, ω-6, cis) and palmitic acid (C16:0), and other bioactive compounds such as vitamin E (α-tocopherol), phytosterols (especially β-sitosterol), and polyphenols (e.g., oleuropein, tyrosol) [32,33,36]. The dietary guidelines of the United States, China, Portugal, and Italy were the only ones that recommend a specific daily intake of oil (27 g/day, <25–30 g/day, 10–30 g/day, and 20–40 mL/day, respectively).

Regarding salt consumption, most selected countries were in line with the WHO recommendations about the maximum daily intake (<5 g/day). The United States suggested a slightly higher amount (<5.75 g/day), whereas China, Sweden, the United Kingdom, and The Netherlands proposed the highest daily intake (<6 g/day). The United States, China, Sweden, France, Italy, and Spain (GENCAT) proposed a maximum daily intake of sugar. Another recommendation provided by some countries (United States, China, Sweden, France, and Spain (GENCAT)) was related to the caloric intake from the consumption of sugar, which should not exceed 10% of the total daily energy intake.

Finally, the consumption of alcohol was particularly limited in the food guides of the United States, China, Sweden, The Netherlands, France, and Italy. Dutch guidelines had the most restrictive limit, with the recommendation of no consumption (or not more than 1 glass/day, equivalent to 10 g of alcohol per day). This is followed by France (not everyone should consume alcohol; <2 glasses/day ≈ 20 g alcohol/day), the United States, Sweden, and Italy (<2 glasses/day in men ≈ 20 g alcohol/day; <1 glass/day in women ≈ 10 g alcohol/day), and finally China with the highest limit (<25 g alcohol/day in men and <15 g alcohol/day in women).

The dietary recommendations for water and other liquids, oil, salt, sugar, and alcohol established by the selected international food guides are included in Table 6.

Based on previous considerations explained throughout the main text, the Scientific Committee of AESAN has updated the dietary recommendations regarding each food group for the Spanish population (Table 7), with the incorporation of portion sizes. According to Almiron-Roig et al., the establishment of adequate food portion sizes is essential to avoid misunderstandings amongst consumers about the amounts of food that should be consumed as well as to decrease their likelihood of overeating [37].

## 4. Conclusions

All the reviewed dietary guidelines provided recommendations for the following food groups: fruits, vegetables, starchy foods (cereals, preferably whole grains, bread, pasta, rice, potatoes), legumes, nuts, milk and dairy products (yogurt, cheese), meat and derivatives, fish, eggs, water, and oil (vegetable oils, most importantly virgin olive oil).

Spanish dietary recommendations proposed by the Scientific Committee of AESAN include a varied and balanced diet characterized by a predominance of plant-based foods and moderate consumption of foods from animal origin. That is, 3–5 servings of fruits/day (it could occasionally be replaced by juice), 2–4 servings of vegetables (raw and cooked)/day, 4–6 servings of cereals/day (preferably whole grains), 2–4 servings of legumes/week, 2–4 servings of milk and dairy products/day, ≥2 servings of fish/week (if possible, 1–2 servings oily fish/week), 2–4 eggs/week, and 2–4 servings of meat/week, preferably chicken or rabbit, and consumption of red meat must not exceed 2 servings/week. Consumption of nuts without added salt as well as virgin olive oil (preferably raw) is recommended as well. Finally, 1.5–2.5 L of water/day should be consumed, and daily intakes of salt and sugar must not exceed 5 and 30 g, respectively.

The present work provided a valuable international overview of twelve food-based dietary guidelines from three different continents (Asia, North America, and Europe). This selection includes dietary guidelines from diverse cultures and traditions directly linked to their eating habits and patterns; however, a more comprehensive comparative analysis including more Food-Based Dietary Guidelines approved in other countries and geographic regions around the world could be addressed in future works based on the results of the present study.

## Figures and Tables

**Table 1:** International overview of Food-Based Dietary Guidelines established in North America (United States), Asia (China), and Europe (Finland, Norway, Sweden, United Kingdom, Germany, The Netherlands, France, Portugal, Italy, and Spain). Adapted from [5,14].

Region/Country	Food-Based Dietary Guidelines	Reference
**North America**		
United States	Dietary guidelines for Americans 2015–2020	[15]
**Asia**		
China	Chinese Dietary Guidelines	[16]
**Europe**		
Finland	Finnish nutrition recommendations 2014	[17]
Norway	Norwegian guidelines on diet, nutrition, and physical activity	[18]
Sweden	Find your way to eat greener, not too much, and to be active!	[19]
United Kingdom	The Balance of Good Health	[20]
Germany	Ten guidelines for wholesome eating and drinking from the German Nutrition Society	[21]
The Netherlands	Dutch dietary guidelines 2015	[22]
France	The French National Nutrition and Health Program’s dietary guidelines	[23]
Portugal	Food wheel guide	[24]
Italy	Dietary Guidelines for Healthy Eating	[25]
Spain	NAOS Strategy.	[26,27]
	GENCAT Strategy (Generalitat de Catalunya)	[28]

**Table 2:** International overview of Food-Based Dietary Guidelines established in North America (United States), Asia (China), and Europe (Finland, Norway, Sweden, United Kingdom, Germany, The Netherlands, France, Portugal, Italy, and Spain). Adapted from [5,14].

Graphic Representations	Country/Region												
	United States	China	Finland	Norway	Sweden	United Kingdom	Germany	The Netherlands	France	Portugal	Italy *	Spain	
												AESAN	GENCAT
Abacus		X											
Key messages (e.g., traffic light)					X			X	X				X
Keyhole symbol					X								
Nutritional circle, oval, or wheel						X	X			X			
Pagoda		X											
Plate	X	X	X										
Pyramid			X									X	

* The Italian guidelines do not include graphical representations. X: information included in the National Dietary Guidelines.

**Table 3:** Food groups and other important categories for which the international food guides have established dietary recommendations.

Food Group	Country/region												
	United States	China	Finland	Norway	Sweden	United Kingdom	Germany	The Netherlands	France	Portugal	Italy	Spain	
												AESAN	GENCAT
Fruits	X	X	X	X	X	X	X	X	X	X	X	X	X
Vegetables	X	X	X	X	X	X	X	X	X	X	X	X	X
Starchy foods	X	X	X	X	X	X	X	X	X	X	X	X	X
Legumes	X	X	X	X	X	X	X	X	X	X	X	X	X
Nuts	X	X	X	X	X	X	X	X	X	X	X	X	X
Soy products	X	X	n.a.	n.a.	-	-	-	-	-	-	-	-	-
Dairy products	X	X	X	X	X	X	X	X	X	X	X	X	X
Meat and its products	X	X	X	X	X	X	X	X	X	X	X	X	X
Fish	X	X	X	X	X	X	X	X	X	X	X	X	X
Seafood	X	-	-	-	X	-	-	-	-	-	X	-	X
Eggs	X	X	X	X	X	X	X	X	X	X	X	X	X
Water and other liquids (tea, etc.)	X	X	X	X	X	X	X	X	X	X	X	X	X
Oil	X	X	X	X	X	X	X	X	X	X	X	X	X
Salt	X	X	X	n.a.	X	X	-	X	-	X	X	X	X
Sugar	X	X	n.a.	n.a.	X	-	-	-	-	-	X	-	X
Alcohol	X	X	n.a.	n.a.	X	-	-	X	X	-	X	-	-
Physical activity	X	X	X	X	X	X	X	X	X	X	X	X	X

X: information included in the National Dietary Guidelines; n.a.: Information not available in the official English version of the National Dietary Guidelines; -: food or food group not mentioned in the National Dietary Guidelines.

**Table 4:** Dietary recommendations for food groups of plant origin established by the international food guides selected in the present work.

Country/Region		Food Groups of Plant Origin					
		Fruits	Vegetables	Starchy Foods	Legumes	Nuts	Soy Products
United States		2 cups-eq/day	2½ cups-eq/day	6 oz-eq/day	1½ cups-eq/week	5 oz-eq/week	
China		200–350 g/day	300–500 g/day (150–250 g of dark green vegetables)	250–400 g/day (50–150 g of whole grains and legumes, 50–100 g of tubers)		-	At least 25 g/day
Finland		>500 g/day (potatoes not included)		-	-	-	-
Norway		At least 5 servings/day		-	-	-	n.a.
Sweden		-	-	-	-	-	-
United Kingdom		At least 5 servings/day		-	-	-	-
Germany		At least 5 servings/day		-	-	-	-
The Netherlands		At least 200 g/day	At least 200 g/day	At least 90 g/day of whole grain bread or other whole grain products	-	At least 15 g/day of unsalted nuts	-
France		At least 5 servings/day (80–100 g/day)		1 portion/meal	-	A handful/day	-
Portugal		3–5 servings/day (1 piece of fruit; medium size = 160 g)	3–5 servings/day (2 cups of raw vegetables = 180 g/day; 1 cup of cooked vegetables = 140 g/day)	4–11 servings/day (1 loaf of bread = 50 g; 1 thin slice of corn bread = 70 g; 1 and 1/2 potato (medium size = 125 g); 5 tablespoons of breakfast cereal = 35 g; 6 biscuits = 35 g; 2 tablespoons of uncooked rice/pasta = 35 g; 4 tablespoons of cooked rice/pasta = 110 g)	1–2 servings/day (1 tablespoon of dried raw legumes (chickpeas, beans, lentils) = 25 g; 3 tablespoons of fresh raw legumes (peas, beans) = 80 g; 3 tablespoons of cooked/fresh legumes = 80 g)	-	-
Italy		2–3 servings/day (1 serving = 150 g)	2½–3 servings/day (1 serving = 200 g)	2½–4½ servings bread/day (1 serving = 50 g)1–1½ servings rice and/or pasta/day (1 serving = 80 g)1½–3 servings breakfast cereals/week (1 serving = 30 g)1–2 servings potatoes/week (1 serving = 200 g)	3 servings/week (1 serving = 150 g)	1–1½ servings/week (1 serving = 30 g)	-
Spain	AESAN	3 servings/day (120 g or a medium piece/a slice of pineapple/two slices melon/watermelon; 150 mL of fresh fruit juice)	2 servings/day (150 g raw in the form of a salad (tomato, lettuce, etc.); 1 serving of sautéed/cooked vegetables as a main course or as garnish of meat, fish or eggs (zucchini, green beans, etc.)	-	At least 2–3 times/week	-	-
	GENCAT	3 servings/day = 1 piece of fruit (1 orange, apple; 2–3 apricots, tangerines, plums, etc.); or 1 bowl cherries, strawberries, grapes; or 1–2 slices of melon, watermelon, pineapple)	2 servings/day (1 dish of cooked vegetables (green beans, puree, stew); 1–2 tomatoes, carrots, cucumbers; 1 pepper, 1 zucchini, 1 eggplant)	-	At least 3–4 times/week	A handful/day; 3–7 handfuls/week	-

n.a.: Information not available in the official English version of the National Dietary Guidelines; -: food or food group not mentioned in the National Dietary Guidelines; c-eq: cup-equivalents; oz-eq = ounce-equivalents.

**Table 5:** Dietary recommendations for food groups of animal origin established by the international food guides selected in the present work.

Country/Region		Food Group of Animal Origin				
		Dairy Products	Meat and Its Products	Fish	Seafood	Eggs
United States		3 cups-eq/day	26 oz-eq/week (meats, poultry, eggs)	-	8 oz-eq/week	26 oz-eq/week (meats, poultry, eggs)
China		300 g of milk/day	280–525 g of poultry or lean meat/week	280–525 g/week	-	280–350 g/week
Finland		5–6 dL of milk/day; 2–3 slices of low-fat cheese	<500 g/week	-	-	-
Norway		-	-	-	-	-
Sweden		-	<500 g/week	-	-	-
United Kingdom		-	-	2 servings/week (at least one of them must be oily fish)	-	-
Germany		-	-	-	-	-
The Netherlands		-	-	1 serving/week (preferably oily fish)	-	-
France		2 servings/day (one serving = 150 mL of milk or 125 g of yogurt or 30 g of cheese)	<500 g/week	-	-	-
Portugal		2–3 servings/day (1 cup of milk = 250 mL; 1 liquid yogurt or 1 and 1/2 solid yogurt = 200 g; 2 thin slices of cheese = 40 g; 1/4 of fresh cheese (medium size) = 50 g; 1/2 curd (medium size) = 100 g)	1.5–4.5 servings/day (raw meat/fish = 30 g; cooked meat/fish = 25 g)		-	1 egg (medium size) = 55 g
Italy		3 servings milk and fermented products/day (1 serving = 125 mL milk, 125 g yogurt)3 servings cheese/week (1 serving = 100 g)	1–3 servings lean meat/week (1 serving = 100 g)1 serving red meat/week (1 serving = 100 g)	2–3 servings/week (1 serving = 150 g of fish including seafood)		2–4 times/week; 1 egg (medium size) = 50 g
Spain	AESAN	-	-	2–4 servings of fish/week (1 serving ≈ 100–125 g of filleted fish or 200–250 g of whole fish (not filleted)	-	-
	GENCAT	1–3 times/day	3–4 times/week (in case of red meat, maximum twice/week)	3–4 servings of fish/week (alternate oily fish with lean fish)	-	3–4 times/week

n.a.: Information not available in the official English version of the National Dietary Guidelines; -: food or food group not mentioned in the National Dietary Guidelines.

**Table 6:** Dietary recommendations for water and other liquids, oil, salt, sugar, and alcohol established by the international food guides selected in the present work.

Country/Region		Water and Other Liquids	Oil	Salt	Sugar	Alcohol
United States		-	27 g/day	5.75 g/day	<10% of the daily energy intake from added sugars	No more than 2 alcoholic drinks/day (men) and 1 alcoholic drink/day (women)
China		7–8 cups of water (1500–1700 mL/day)	<25–30 g/day	<6 g/day	<50 g/day; <10% of the daily energy intake	<25 g/day (men); <15 g/day (women)
Finland		-	-	<5 g/day	n.a.	n.a.
Norway		-	-	n.a.	n.a.	n.a.
Sweden		-	-	<6 g/day	<10% of the daily energy intake	<20 g/day (men); <10 g/day (women)
United Kingdom		6–8 glasses or cups of water/day	-	<6 g/day	-	-
Germany		At least 1.5 L/day	-	-	-	-
The Netherlands		3 cups of tea/day	-	<6 g/day	-	Do not consume alcohol or no more than 1 glass/day (1 glass ≈ 10 g of alcohol; 250 mL of beer ≈ 5% alcohol, 100 mL of wine ≈ 12% alcohol; 35 mL of alcohol ≈ 35% alcohol)
France		-	-	-	-	No more than 2 glasses/day and not everyday
Portugal		-	1–3 servings/day (1 tablespoon of olive oil/oil = 10 g; 1 teaspoon of lard = 10 g; 4 tablespoons of cream = 30 mL; 1 tablespoon of butter/margarine = 15 g)	<5 g/day	-	-
Italy		1.2–2 L/day (at least 6–10 glasses of water)	2–4 servings/day (1 serving = 10 mL olive and/or vegetable oil)	<5 g/day	5–10 g/day	No more than 2 alcoholic drinks/day (men) and 1 alcoholic drink/day (women)
Spain	AESAN	At least 1.5 L/day (5–8 glasses of water or other liquids/day)	-	<5 g/day	-	-
	GENCAT	-	-	<5 g/day	<10% of the daily energy intake	-

n.a.: Information not available in the official English version of the National Dietary Guidelines; -: food or food group not mentioned in the National Dietary Guidelines.

**Table 7:** Dietary recommendations for each food group and other important categories proposed by the Scientific Committee of AESAN. Adapted from [5].

Food Group	Frequency of Consumption	Weight of Each Serving
Fruits	3–5 servings/day Sporadically substitute for juice	120–200 g of fresh fruit 150 mL of juice
Vegetables	2–4 servings/day Combine different products (raw and cooked)	150–200 g
Starchy foods (preferably whole grains)	Daily consumption 4–6 servings/day	40–60 g of bread
		60–80 g of pasta, rice
Legumes	2–4 servings/week	50–60 g
Nuts	Weekly, several times	20–30 g No salt added
Dairy products	Daily consumption 2–4 servings/day	200–250 mL of milk
		80–125 g of fresh cheese 40–60 g of mature cheese
		125 g of yogurt, and other fermented milks, with no added sugar
Meat and its products	2–4 servings/week Preferably chicken or rabbit meat No more than 2 servings of red meat/week	100–125 g
Fish/seafood	At least 2 servings/week 1–2 servings of oily fish/week	125–150 g
Eggs	2–4 eggs/week	Medium size (53–63 g)
Water	1.5–2.5 L/day	200–250 mL
Virgin olive oil	Daily consumption Preferably raw	10 mL
Salt	<5 g salt/day = 2 g sodium/day Do not add during cooking Avoid food with added salt	-
Sugar	<30 g/day Avoid foods with added sugar	5–10 g

## Data Availability

Not applicable.
